# Communication style drives emergent leadership attribution in virtual teams

**DOI:** 10.3389/fpsyg.2023.1095131

**Published:** 2023-03-24

**Authors:** Scott M. Rennie, Lana Prieur, Michael Platt

**Affiliations:** ^1^Department of Neuroscience, University of Pennsylvania, Philadelphia, PA, United States; ^2^Department of Marketing, University of Pennsylvania, Philadelphia, PA, United States; ^3^Department of Psychology, University of Pennsylvania, Philadelphia, PA, United States

**Keywords:** leadership selection, virtual teams, synchrony, speech analysis, facial affect, group decision making

## Abstract

Leader selection plays a key role in how human social groups are formed and maintained. Leadership is either assigned through formal processes within an organization, or emerges informally through interactions with other group members–particularly in novel contexts. COVID-19 has accelerated the adoption of virtual meetings and more flexible team structures. However our understanding of how assigned leadership influences subsequent leadership emergence in virtual settings is limited. Here we examine the relationship between assigned leadership within an existing organization and subsequent emergent leadership attributions as members engage in virtual interactions. To do so, we created and implemented a novel virtual group decision-making task designed to support quantification of a more comprehensive set of communication style elements, such as speech dynamics and facial expressions, as well as task behaviors. Sixteen members of a real world organization engaged four repeated rounds of a group decision making task with new team members each time. We found participants made novel attributions of emergent leadership rather than relying solely on existing assigned leadership. While assigned leadership did influence leadership attributions, communication style, including amount of speech but also variability in facial expressions, played a larger role. The behavior of these novel emergent leaders was also more consistent with expectations of leadership behavior: they spoke earlier, more often, and focused more on the correct decision than did assigned leaders. These findings suggest that, even within existing social networks, virtual contexts promote flexible group structures that depend more on communication style and task performance than assigned leadership.

## Introduction

Forming groups is a universal feature of human social behavior (Gordon et al., 2014; Shamay-Tsoory et al., 2019). How we attribute leadership status to others is a key aspect of how we form and manage those groups (Spisak et al., 2015). Broadly speaking, leadership attribution occurs either by (1) assigning it through formal processes within an organization or (2) by it emerging informally through the impressions of other group members (Hollander, 1960; Stein and Heller, 1979; Paunova, 2015).

Early research argued that leadership depended upon traits inherent to individuals (Lewis, 2000), which explain some variance in whether individuals emerge as a leader. However, leadership emergence is increasingly considered to be a relational process, depending more upon the dynamics of verbal and non-verbal communication in groups than upon the distribution of individual traits (DeRue and Ashford, 2010; Hackman and Johnson, 2013; Gerpott et al., 2018). Much of the work on this topic has nonetheless remained focused on traits and other static antecedents of emergent leadership (Gerpott et al., 2018) rather than the relationship between assigned and emergent leadership over time, or to the dynamics of behaviors that drive attributions of emergent leadership in groups.

Communication style, particularly speech, is considered so central to leadership attribution that leadership has been referred to as a “language game” (Pondy, 1989). Speech, focused on task outcomes reliably predicts emergent leadership (Bales, 1950; Lonetto and Williams, 1974) particularly when related to information-seeking and sharing (Morris and Hackman, 1969). The tendency to participate in discussion early and regularly has been shown in numerous studies to predict perceptions of emergent leadership (Morris and Hackman, 1969; Sorrentino and Boutillier, 1975; Kickul and Neuman, 2000; Sudweeks and Simoff, 2005).

The display and management of emotions through both verbal and non-verbal communication (Adolphs, 2001; Kelly and Barsade, 2001; Barsade and Gibson, 2012) drive perceptions of leadership (Pescosolido, 2002; Li et al., 2012). Facial expressions are a primary means of signaling emotions, one for which we have evolved unique facial musculature (Ekman, 1993). Links between facial expressions, particularly emotional signaling, and perceptions of leadership have a well-developed theoretical basis (Trichas et al., 2017; Trichas, 2017a). Emotional signaling plays an important role in group coordination (Cosmides and Tooby, 2000), influencing a person’s perception in dimensions relevant to leadership attributions (Zebrowitz and Montepare, 2008; Trichas, 2017b). For example, the variability of an individual’s facial expressions has been shown to have a positive influence on perceptions of their leadership, authenticity and trustworthiness (Slepian and Carr, 2019).

Our ability to synchronize behavior with others is both a measure and a mediator of dyadic and group communication and cohesion (Wiltermuth and Heath, 2009; Dikker et al., 2017; Gordon et al., 2020). Facial mimicry–the synchronization of facial expressions–is thought to support social cohesion through enhancing emotional recognition (Fischer and Hess, 2017; Slepian and Carr, 2019), affective and cognitive empathy (Hofelich and Preston, 2012; Drimalla et al., 2019; Holland et al., 2021) and perspective-taking (Stel et al., 2008). Inhibiting facial mimicry reduces credibility and cognitive empathy (Ask, 2018). Perspective-taking, emotional recognition, and empathy are all thought to contribute to emergent leadership attributions (Pescosolido, 2002; Wolff et al., 2002).

A comprehensive account of the leadership attribution process requires quantifying the rich array of dynamic social cues, diverse communication styles, and competencies that constitute human group behavior (Hollander, 1960; Stein and Heller, 1979; Pondy, 1989; Gerpott et al., 2018). Development of computational approaches to quantifying key aspects of communication, such as facial expressions and speech dynamics, and various forms of interpersonal synchrony, has been developing rapidly. However these make up only a subset of the rich multi-dimensional content of social interactions, and are best studied in tightly controlled contexts, thus making the comprehensive quantification of real-world human group interactions notoriously difficult. This challenge emphasizes a tension between realistic, ecologically valid experiments, where much of the richness of group interactions is maintained, and the ability to maintain control over experimental variables and their quantification. This tension tends to result in approaches that either use abstract, simplified paradigms where richness and ecological validity is sacrificed for tight experimental control, or realistic group interactions with low experimental control and many crucial variables remaining unquantified.

Teleconferencing constrains the richness of social interactions in shared physical space, yet much of that richness is maintained (Werkhoven et al., 2001). Indeed, despite reductions in eye contact, obscured expression of postural and gestural cues, and of course physical proximity, teleconferencing has shown only limited or conflicting impacts on measures of group task performance (Sellen, 1995; McLeod et al., 1997; Brucks and Levav, 2022). Crucially, as teleconferencing requires that the entirety of a social interaction including the information each individual sends and receives is mediated by software, it becomes much more amenable to comprehensive quantification.

Teleconferencing is also becoming increasingly common in personal and professional interactions, and abruptly increased during the COVID-19 pandemic (Frost and Duan, 2020; Arrero et al., 2021; Chai and Park, 2022) It has been estimated that as much as 20% of all United States workdays will continue remotely once the pandemic has passed (Barrero et al., 2021; Brucks and Levav, 2022). Teleconferencing therefore provides not only a powerful way of quantifying social interactions but is also becoming an increasingly common form of everyday human interaction.

The proliferation of the use of teleconferencing, the richness of virtual social interactions, and their increased amenability to quantification provide a unique balance between maintaining experimental control and ecological validity. This makes it an ideal tool for studying realistic human group behavior and leadership selection.

While the literature on leadership is extensive, research focusing on leadership, and specifically leadership selection in virtual interactions, is limited but growing (Kayworth and Leidner, 2002). Much like in-person teams, leaders regularly emerge through contribution and influences upon the team (Yoo and Alavi, 2004) and often virtual teams have greater shared leadership (Frost and Duan, 2020). It is thought that the lack of shared physical space can undermine the effectiveness of group communication (McDonough et al., 2001; Frost and Duan, 2020). Typical social skills involved in in-person leadership may not be enough to lead in the online environment (Roman et al., 2019), rather, additional skills in communication are required (Contreras et al., 2020). Therefore, communication style, specifically frequency, quality and quantity of communication, has been hypothesized to be more important for leadership in virtual teams (Kayworth and Leidner, 2002; Kirkman et al., 2004; Yoo and Alavi, 2004).

Here we focus on an important but overlooked aspect of leadership: how existing assigned leadership impacts emergent leadership attributions in virtual interactions. We examined the effect of hierarchical standing by conducting a teleconferencing group decision making task with members of an established professional network with pre-assigned leadership roles. We compared the influence of established assigned and dynamic in-task variables—communication style, speech and facial expressions, and task performance have on subsequent leadership attribution. We then modeled how assigned leadership, task behaviors, and subjective ratings predicted actual leadership selection. We found that participants made novel attributions of emergent leadership rather than deferring to the assigned leadership defined by their professional network. While assigned leadership did influence leadership attributions it was less predictive of leadership attribution than elements of communication style generally and the amount of time spent speaking in general. The behavior of these novel emergent leaders was also more consistent with expectations of leadership behavior than that of assigned leaders; they spoke earlier, more often, and focused more on the correct task outcome. This indicates that while participants were attributing leadership with greater reliance on communication style than assigned leadership they did so in a way that was guided by both competence and leader-like behaviors. This suggests that teleconferencing may support more flexible status structures and teams that are able to assess leadership in an effective manner both of which could support increasingly common, dynamic teams.

## Materials and methods

### Participants

During the summer of 2020 we recruited 16 University of Pennsylvania undergraduate students (M age = 20 SD age 1.31) who were all part of the same social network—a medical fraternity club— and repeated the experiment 4 times. Because all participants were part of a club that included leadership positions, each participants’ club status was manually coded with a larger number denoting a higher status (e.g., president of the club: 9) and a lower number denoting a lower status (e.g., a freshman at entry level in the club: 1). All members take part in frequent shared club activities, and hold different explicit roles that vary in status. All participants had normal or corrected to normal vision. Written and informed consent was obtained from each participant prior to the experiment, and confirmed on each subsequent experimental session. The study procedures were approved by the University of Pennsylvania Institutional Review Board.

### Hidden profile task

**Figure 1:**
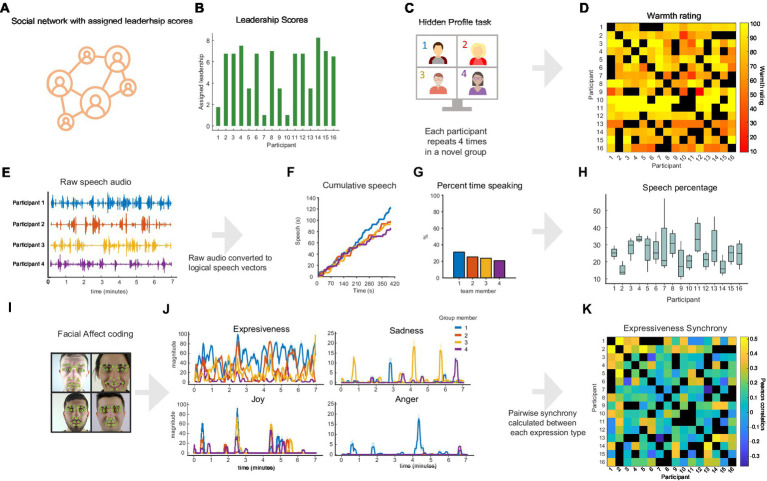
Experimental approach and analysis pipeline: **(A)** Quantified social network, **(B)** Leadership scores of participants in social network. **(C)** Hidden profile task schema. **(D)** Ratings of participants on warmth and competence from other participants. **(E–G)** Example data from session 10, **(E)** Raw speech waveforms **(F)** cumulative speech over session durations **(G)** Percentage of total speech by each participant. **(H)** Percentage of speech by each participant in each session. **(I)** Facial affect coding shema. **(J)** Example facial affect time course from session 10, Engagement, Joy, Sadness and Anger **(K)** pairwise synchrony between interacting participants, black squares indicate participants that did not interact. Faces created with AI software (https://stablediffusionweb.com/) License: CC0 1.0 Universal Public Domain Dedication.

Each participant engaged in four rounds of a hidden profile task (HPT) as a means to measure both individual and group performance in information-sharing and consensus-seeking. The HPT is designed so that the optimal outcome can only be explicitly uncovered when group members collectively pool information that they acquire individually before the task begins. Some of this information is shared among all members, but, crucially, some information is uniquely held by each of them. Each participant engaged in four hidden profile task discussions in groups of four (Figure 1C). We pseudo-randomly selected participants to maximize the number of novel pairings and avoid repeat pairings.

Prior to the discussion each participant is given a short vignette that contains information on three choices available to them, crucially some of that information is shared across all participants while some information is hidden from all but one participant. The optimal decision can only be reliably reached by the sharing of the hidden information. Small groups engaging in a HPT tend to have a collective information sampling bias toward basing their decision on commonly-held rather than unique information, leading to suboptimal outcomes (Stasser and Titus, 1985, 1987; Stasser and Stewart, 1992). The commonly-held information is designed to favor an incorrect choice while the unique information held by individuals supports the correct option and invalidates the incorrect ones.

Participants were given 10 min to read the assigned vignette containing the shared and hidden information units, they were then instructed to reach a consensus and given 10 min to do so. They were prompted 2 and 1 min before this time elapsed, and whether the group reached consensus, and which choice the group made was then recorded. The HPT relies on distribution of hidden information, and classically on the participants being unaware that they have access to information that other members do not. Having participants engage in repeated HPT’s did not lead to significant changes in group behavior—i.e., coordinating to explicitly announce all information each participant had at the start of a session, rather than revealing information through discussion.

### Pre HPT measures

Before their first group discussion, each participant completed measures of their demographics and individual differences, including anxiety. Each participant also completed the anxiety measure before their second, third, and fourth rounds. Trait and state anxiety were measured using the State–Trait anxiety index (Spielberger, 2012), Grit was measured using the short Grit Scale (Duckworth and Quinn, 2009), Generalized Problematic Internet Usage Scale-2 (GPIUS2; Caplan, 2010) and Interpersonal Reactivity Index (IRI; Davis, 1980).

### Post HPT measures

Following the HPT, participants were asked to choose which participant they considered the leader and rate that participant on a modified Multifactor Leadership Questionnaire 6S (MLQ-6S) and rate both themselves and each of the other participants in the experimental group on warmth and competence. Following completion of these measures, participants were informed as to whether they had chosen the correct outcome or not.

### Audio analysis

We made use of the native recording options in Zoom to obtain a separate audio track for each participant, and a single audio track for the whole group, per session (Figure 1E). Each participant’s audio track was processed in MATLAB and converted to logical speech/silence vectors. These speech vectors were then used to calculate quantity, timing and dynamics of speech (Figures 1F,H). One participant’s audio was excluded due to a recording error.

### Facial expressions and mimicry

Each video session was cropped into individual videos using a simple function in Bonsai (Lopes et al., 2015). These videos were then imported in iMotions Biometric Research Platform 9.0 software and analyzed using the Affectiva Facial expression recognition engine (Bishay et al., 2022; Figures 1I,J). To be clear, and in line with a growing body of research, we do not consider affect expressed in facial expressions as veridical measures of a participant’s affective state (Kappas, 2003; Mauss and Robinson, 2009; Yang, 2014). We instead consider it to be a measure of emotional signaling, while remaining agnostic to the participant’s internal affective state. The mean and standard deviation for each emotion category was calculated for each participant in each session. As we were quantifying mimicry of macro-expressions, which tend to vary between 0.5–4 s (Rosenberg and Ekman, 2020), and to reduce the role of false positives facial expression data was smoothed with a 2 s before conducting pairwise Pearson correlations between each participant in each group (Figure 1H).

### Modeling description

Here we examined whether pairwise emergent leadership choices could be predicted by key task variables. As participants engaged in four HPT sessions with three other participants each time, this provided 192 individual leadership choices. We initially included the following trait predictors: assigned leadership score, trait anxiety, grit and gender; the task predictors: amount of information conveyed, a conveyed information “unit” was defined as an outcome relevant element of the HPT vignette spoken by a participant. We included the following communication style predictors: mean facial expression magnitude for each emotion category, valence and expressiveness, and the proportion of time the potential leader spent speaking. We included expression mimicry predictors: the pairwise Pearson correlation between potential leader and participant in both expressiveness and valence categories. Finally, we included pairwise participant ratings of warmth and competence. We used a generalized linear model with a logit link function, random intercept and added and removed variables in a backwards stepwise manner using a Bayesian Information Criterion implemented using the *glmfit.m* and *stepwiseglm.m* functions from the statistic toolbox in MATLAB (2022; The Mathworks, Inc., Massachusetts).

## Results

### Group performance

**Figure 2:**
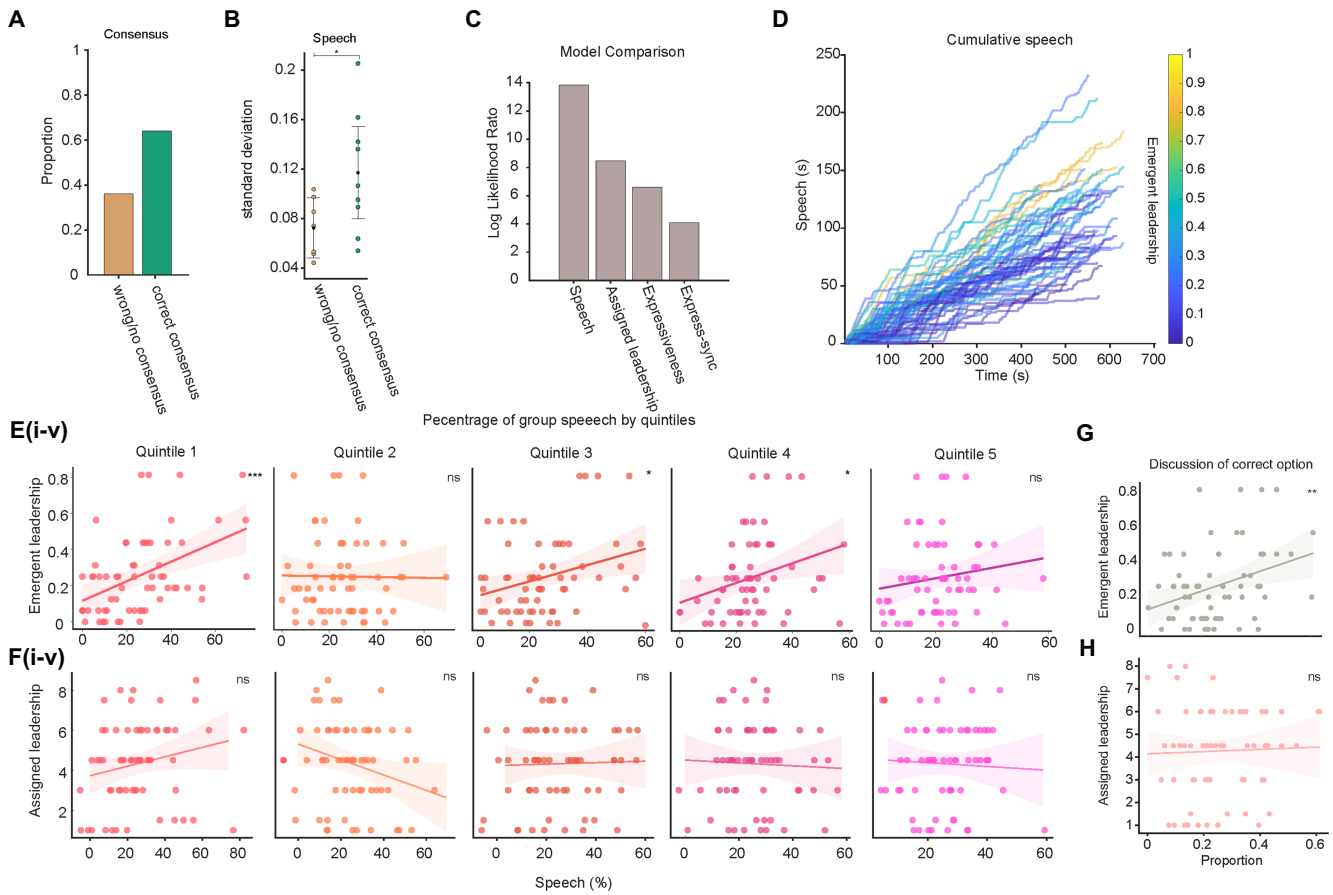
Communication style predicts emergent leadership attribution. **(A)** Proportion of 16 sessions that reached the correct consensus. **(B)** Standard deviation of speech percentage between correct consensus and incorrect groups. **(C)** Log likelihood ratio of reduced models compared to best models. **(D)** Cumulative speech for each participant session color temperature indicates emergent leadership score. **(E,F)** Relationship between percentage of speech and **(E)** emergent or **(F)** assigned leadership by each participant in a session split into five (i–v) quintiles. **(F)** Percentage of speech and assigned leadership proportion by each participant in a session split into five quintiles. **(G,H)** Relationship between each proportion of each group’s discussion of the correct outcome by each participant and emergent and assigned leadership.

Groups reached the correct consensus in 10 of 16 HPT sessions; in the remaining sessions they failed to reach consensus in 2 and reached an incorrect consensus in 4 (Figure 2A). There are three primary measures that determine how information is being used in a HPT: (1) the proportion of total available hidden information shared; (2) information pooling, the percentage of unique information mentioned out of total information available; and (3) discussion focus, the percentage of unique information out of total information discussed (Lu et al., 2012). We found no significant differences in any of these three measures between groups that succeeded or failed to reach consensus. However, when examining the distribution of speech between groups, we found that the correct consensus groups had greater dispersion in the quantity of speech across participants (*T*_14_ = 2.1804, *p* < 0.05; Figure 2B). This suggests that the tendency for particular individuals to lead the discussion more than others, rather than information sharing, played a greater role in groups reaching the correct outcome.

### What determines emergent leadership selection?

Our primary research question focuses on what qualities drive the selection of individuals leaders. To answer this question, we fit a generalized linear model to predict whether a participant would choose another participant as the group leader. Each participant rated and was rated by 12 of the 16 other participants across four separate sessions. To remove non-significant predictors, we added and removed variables in a backward stepwise manner using a Bayesian Information Criterion (BIC). We initially included the following trait predictors: assigned leadership score, trait anxiety; and grit. We included task predictors: the amount of information, with each sentence quantified as a conveyed ‘information unit’. We included facial affect and mimicry predictors: the mean magnitude and pairwise Pearson correlation between each participant and all other members of their group in expressiveness—the variability in expressions across all categories, and valence—the net magnitude of positive and negative expressions and the proportion of time the potential leader spent speaking. Finally, we included pairwise participant ratings of warmth and competence.

The final model significantly predicted leadership choice [*X*^2^(184, *N* = 189) = 40.5, *p* < 0.0001]. Variables that survived exclusion were predominantly related to communication style: proportion of time speaking, median expressiveness and expressiveness mimicry as did assigned leadership. We found that speech proportion, average expressiveness during an individual’s speech, and real world status each positively predicted leadership emergence while expressiveness entrainment was a weak negative predictor (Table 1).

**Table 1:** Leadership selection model.

Predictor	Estimate	Standard Error	T stat	*P-V*alue
Intercept	−5.3485	0.94581	−5.6549	<0.0001
Speech percentage	7.181	2.0778	3.4561	<0.0001
Assigned leadership	0.27561	0.10235	2.6928	<0.001
Expressiveness	0.032582	0.013088	2.4894	0.01
Expressiveness mimicry	−1.9705	0.94681	−1.9843	<0.05

To more precisely quantify the importance of each surviving predictor we performed a series of model comparisons, with a leave one out approach, and compared the log likelihood ratio of each reduced model to the full model (Figure 2A). As expected speech percentage was the most important predictor, and yielded the greatest decrease in log-likelihood ratio, followed by assigned leadership, mean expressiveness and expressiveness synchrony. This demonstrates that while assigned leadership did influence emergent leadership attribution, communication style generally, and proportion of speech specifically, played a much more important role.

### Speech qualities are related to emergent but not assigned leadership

Speech quantity was the greatest predictor of emergent leadership attribution. We therefore quantified which elements of speech had the strongest relationship to both emergent and assigned leadership. To facilitate the comparison between emergent and assigned leadership, we calculated an emergent leadership score as the proportion of the total possible times each individual was chosen as a leader.

We visualized speech dynamics by calculating the cumulative sum of speech for each participant (Figure 2B). This illustrated that individuals with higher emergent leadership contributed both more and earlier to each discussion. Leaders have a tendency to speak more, earlier (ref). We therefore quantified these patterns by splitting each session into quintiles and plotting each participant’s speech against both their emergent and assigned leadership scores. We found that emergent leadership most strongly predicted the percentage of speech in the first quintile [*F*(63,61) = 16.2 *p <* 0.001] with an *R^2^* of 0.194, and weaker positive relationships in the 3rd [*F*(63,61) = 6.16 *p <* 0.05, *R^2^* = 0.09] and 4th [*F*(63,61) = 5.97 *p <* 0.05, *R^2^* = 0.0892] quintiles. Assigned leadership failed to significantly predict the percentage of speech in any quintile. This indicates that emergent leaders spoke earlier, initiating conversation, but did not proceed to dominate it throughout the session, while assigned leaders’ behavior was not distinguishable from other group members.

A benefit of the HPT is that it allows, in addition to quantifying the amount and timing of speech, to quantify elements of competence in task focused discussion. Because the HPT has a correct solution, which the group must reach consensus upon for success, competence can be measured by the proportion time each participant spends discussing the correct vs. incorrect options (Figures 2G,H). Two linear regressions were calculated to predict time spent speaking about the correct option from emergent and assigned leadership. A significant regression equation was found for emergent leadership [*F*(64,62) = 8.52 *p* < 0.01] with an *R^2^* of 0.121, but not assigned leadership, [*F*(64,62) = 0.001 *p* = 0.07] with an *R^2^* of 0.0017. Thus, emergent leaders displayed more competence in their task-focused communication than participants with higher assigned leadership. This finding suggests that participants made effective choices when choosing group members as emergent leaders rather than relying on assigned leadership status.

## Discussion

Multiple studies have linked communication style, particularly the quantity and timing of speech, to emergent leadership attribution (Morris and Hackman, 1969; Sorrentino and Boutillier, 1975; Kickul and Neuman, 2000; Sudweeks and Simoff, 2005). Here we predict leadership attribution from a range of communication style elements, including speech dynamics, information content of speech and facial expressions within members of a professional social network with an established assigned leadership structure. By using a novel approach, examining teleconferencing interactions in combination with a HPT, we were able to combine high experimental control with high ecological validity and quantify a more comprehensive range of communication style elements and task relevant information conveyed by each participant.

Our primary research question focused on how existing assigned leadership within a social network would influence emergent leadership attribution in a teleconferencing context. Two expectations were that (1) if assigned leadership plays the same role in a teleconferencing as it does in face to face interactions it would be most predictive of leadership attribution and (2) that the behavior of assigned leaders would be most consistent with established leadership behaviors. In contrast, our model of leadership attribution demonstrated that the quantity of speech in particular, and communication style as whole, were better predictors of leadership attribution than previously-assigned leadership, which validate previous studies (Pescosolido, 2002; Zebrowitz and Montepare, 2008; Li et al., 2012; Trichas, 2017a; Slepian and Carr, 2019).

The medium of teleconferencing has been shown to reduce the impact of existing team identity (Joshi and Roh, 2009). We speculate that this might extend to decreasing the importance of assigned leadership within a particular social network. Particularly, changes in information sharing dynamics in digital settings offer the possibility of flatter hierarchies, more flexible leadership roles (Cortellazzo et al., 2019) and greater performance. In line with this we also found that novel emergent leaders, but not assigned leaders, demonstrated patterns of communication that are classically associated with leadership, and largely consistent with a dominant leadership style (D’Errico and Poggi, 2019). They spoke earlier, engaged in more competent, task-focused communication, and increased discussion of the correct outcome. Participants therefore did not rely exclusively upon assigned leadership and instead integrated it with group members’ communication style and task behavior.

The existing research on various aspects of team effectiveness during teleconferencing is mixed. These data argue that leadership attribution is more flexible being be more driven by accurate appraisals of current social information in teleconferencing contexts. Information shared among all members is crucial to group performance in the HPT. A flatter hierarchy, in this example with less reliance on existing assigned leadership, may allow for more a wider distribution of information contributions from the group (Cortellazzo et al., 2019) which may result in better performance for virtual groups (Pearce et al., 2009; Hoch and Kozlowski, 2014).

Emotional regulation is thought to be an important predictor of leadership attribution (Pescosolido, 2002; Li et al., 2012). Anxiety represents a maladaptive form of emotion regulation (Cisler and Olatunji, 2012), while grit is thought to be related to successful emotional regulation and perseverance (Hwang and Nam, 2021). Perspective-taking ability is thought to support leadership through detection and management of group emotion and task relevant information (Wolff et al., 2002). We found that none of these traits had any measurable impact on leadership attribution. These findings underscore the relational nature of leadership attribution over the role of specific individual traits.

Neither the average magnitude of signaled emotion, nor pairwise synchrony in any of these categories, predicted leadership attribution. We note that the HPT vignettes used in this study were more procedural and thus unlikely to evoke strong emotional responses, thus making the average magnitude of emotional expression less informative. Nonetheless, consistent with the findings of Slepian and Carr (2019) we found that the variability in expressiveness rather than the valence of signaled emotion positively predicted leadership attribution. This diminished role of signaled emotion synchrony, despite being considered important for group behavior and leadership selection, may reflect disruption of social presence and interpersonal synchrony thought to be caused by virtual interactions (Gutman et al., 2022). Expressiveness synchrony negatively predicted emergent leadership attribution, this may be the result of participants attempting to attract the attention of the group at the same time. Interpersonal competition has been found to negatively predict leadership attribution between individuals sharing individual differences such as social dominance (D’Errico, 2020). Expressiveness synchrony may also be an indication of competition in this data.

### Limitations

A limitation of this study is that it focuses on a single social network, an undergraduate medical fraternity, which limits the extent to which these findings can generalize to other organizational contexts. While undergraduate medical fraternities are professional and have clear assigned leadership positions, they may be treated differently from more formal and longer lasting professional networks. This may limit the impact of assigned leadership.

There is evidence that virtual interactions reduce the impact of hierarchical status (Joshi and Roh, 2009; Lilian, 2014). Nonetheless this study does not aim to provide a quantified comparison of the relative importance of assigned leadership, communication style and task behavior between in-person and teleconferencing leadership attribution. Further research explicitly comparing in person and online interactions would be required to address this question explicitly.

### Conclusion

The rise in the use of virtual communications is coinciding with an increasing reliance on more flexible and dynamic teams (Lilian, 2014) challenging traditional hierarchical structures. This study demonstrated a novel paradigm that can be used to quantify a more comprehensive account of the milieu of social cues that make up group behavior. We implemented this new technique finding that virtual teams are able to effectively integrate assigned leadership with the communication style and task behavior of group members when making leadership attributions. Additionally, the virtual setting, as opposed to an in-person one, may allow individuals to emerge as more effective leaders than those formally assigned, supporting both more agile and flexible teams and potentially increasing status mobility.

## Data availability statement

The raw data supporting the conclusions of this article will be made available by the authors, without undue reservation.

## Ethics statement

The studies involving human participants were reviewed and approved by University of Pennsylvania Institutional Review Board. The patients/participants provided their written informed consent to participate in this study.

## Author contributions

SR designed the experiment, conducted data analysis, and wrote the paper. LP conducted data collection and took part in both experimental design, analysis, and writing. MP is the lead investigator of the lab who carried out the study. All authors contributed to the article and approved the submitted version.

## Funding

Funding was provided by grant R01-MH108627 and a Wharton Dean’s Postdoctoral Fellowship (SR).

## Conflict of interest

The authors declare that the research was conducted in the absence of any commercial or financial relationships that could be construed as a potential conflict of interest.

## Publisher’s note

All claims expressed in this article are solely those of the authors and do not necessarily represent those of their affiliated organizations, or those of the publisher, the editors and the reviewers. Any product that may be evaluated in this article, or claim that may be made by its manufacturer, is not guaranteed or endorsed by the publisher.
